# Discrimination between Alternative Herbal Medicines from Different Categories with the Electronic Nose

**DOI:** 10.3390/s18092936

**Published:** 2018-09-04

**Authors:** Xianghao Zhan, Xiaoqing Guan, Rumeng Wu, Zhan Wang, You Wang, Guang Li

**Affiliations:** State Key Laboratory of Industrial Control Technology, Institute of Cyber-Systems and Control, Zhejiang University, Hangzhou 310027, China; 3150105206@zju.edu.cn (X.Z.); 3150103730@zju.edu.cn (X.G.); 3150102424@zju.edu.cn (R.W.); 11732003@zju.edu.cn (Z.W.); king_wy@zju.edu.cn (Y.W.)

**Keywords:** conformal prediction, electronic nose, herbal medicine, support vector machine, reliability

## Abstract

As alternative herbal medicine gains soar in popularity around the world, it is necessary to apply a fast and convenient means for classifying and evaluating herbal medicines. In this work, an electronic nose system with seven classification algorithms is used to discriminate between 12 categories of herbal medicines. The results show that these herbal medicines can be successfully classified, with support vector machine (SVM) and linear discriminant analysis (LDA) outperforming other algorithms in terms of accuracy. When principal component analysis (PCA) is used to lower the number of dimensions, the time cost for classification can be reduced while the data is visualized. Afterwards, conformal predictions based on 1NN (1-Nearest Neighbor) and 3NN (3-Nearest Neighbor) (CP-1NN and CP-3NN) are introduced. CP-1NN and CP-3NN provide additional, yet significant and reliable, information by giving the confidence and credibility associated with each prediction without sacrificing of accuracy. This research provides insight into the construction of a herbal medicine flavor library and gives methods and reference for future works.

## 1. Introduction

Following its long history, alternative herbal medicine has gained popularity across the world [1]. However, its classification tends to be a difficult job [2,3]. There is an idiosyncrasy associated with herbal medicines—many different categories of herbal medicines have similar physical appearances after preprocessing in pharmacies. Meanwhile, the most convenient means for discrimination of herbal medicines currently is to consult doctors which is highly dependent on doctors’ knowledge and experience. Therefore, the similarity between herbs’ appearance necessitates a large amount of time for discrimination and elicits high error rates, leading to certain merchants’ substituting expensive medicines for inferior counterparts. Therefore, it is necessary to put forth a standard and convenient quality evaluation process to distinguish between herbal medicines of different categories for the betterment of customers all around the world.

The artificial olfaction system, generally recognized as electronic nose, simulates the mechanism of mammal olfaction and can be used as a fast, cheap, non-instrumental, and stable analytical tool to deal with mixtures of volatile compound gas. So far, electronic nose has been applied in such domains as evaluating environment quality [4,5,6,7,8], medical diagnosis [9,10,11,12,13,14], and especially, in food evaluation [15,16,17]. With regard to food evaluation, Wojnowski and his team [18] used a portable modular electronic nose intended for food analysis, combined with the SVM method, to successfully classify poultry and rapeseed oil samples. The prototype was also used to detect the adulteration of extra virgin olive oil and rapeseed oil with an overall accuracy of 82%. In the article “Electronic noses for food quality: A review” [19], the authors summarize recent work related to electronic nose use in the food industry, focusing on the application of the electronic nose in food quality monitoring, such as in meat, milk, fish, tea, coffee, and wine. Macías Miguel et al. [20] developed a portable and low-cost electronic nose prototype based on an mbed microcontroller and tested the performance of the electronic nose by measuring the ethanol content of the wine synthetic matrix. The electronic nose with a neural network classifier is used to distinguish wine samples containing 10%, 12%, and 14% V/V alcohol content with a classification error of less than 1%. Majchrzak, T. and co-workers [21] have successfully used electronic noses for the analysis of edible oils, in particular, for determining the geographical origins of products, as well as for detecting adulteration and deterioration caused by external factors. Additionally, the group led by Lin [22] used the electronic nose to analyze the juices of raw lotus root (RLR) and full lotus root powder (FLRP) and found the homogeneity of their major olfactory components. Rodriguez and colleagues [23] analyzed the quality of coffee with the E-nose and classified the flaws of Columbian coffee to justify the efficacy of using the E-nose to evaluate coffee quality. Li and co-workers [24] integrated the E-nose and chemistry analytical method to distinguish red ginsengs from Korean ginsengs. By implementing Principal Component Analysis (PCA), Discriminatory Factor Analysis (DFA), and Soft Independent Model Cluster Analysis (SIMCA), this group successfully classified these two kinds of ginsengs. Miao and his co-workers [25] combined the results from the E-nose and NIR (Near Infrared) to successfully distinguish ginsengs from nine different locations. By using the support vector machine, these scholars reached classification accuracies of 90.18±4.94% and 97.98±2.77% for the E-nose and NIR respectively. Afterwards, with data fusion methods at the feature level and decision level, the accuracies were shown to be 99.58±1.23% and 99.24±1.57%, indicating that data fusion can be used to expand the results gained from electronic nose.

In addition to classification results, the reliability of classifications of alternative herbal medicine is also of great significance, since the results are closely associated with the treatment of diseases. A bad classification with poor reliability renders the discrimination process useless and poses direct threats to patients’ health. Currently, in order to provide information about the prediction reliability, methods such as probably approximately correct learning (PAC) and Bayesian learning have been issued. Nevertheless, the large number of samples that do not offer details about the reliability of individual prediction [26] lead PAC to be not so appropriate for the herbal medicine classification problem. On the other hand, although methods such as Bayesian learning, logistic regression [27], and Platt’s method [28] do associate individual prediction with additional reliability information, they are usually based on stringent distribution assumptions. Notwithstanding, since data gathered from the E-nose is usually influenced by sensor drifts due the variations in the surrounding environment, the distribution assumptions cannot be readily satisfied.

Conformal prediction was issued and improved by Vladimir Vovk and his co-workers [26,27,29,30]. It is based on the identical and independent distribution assumptions which state that all samples and labels are generated from the same identical and independent distributions which is a weaker assumption when compared with the methods mentioned above. When applied to the processing of E-nose data, conformal prediction can provide promising information about reliability for each prediction. With the characteristic nonconformity measure, conformal prediction can provide additional information about confidence and credibility for each prediction made and avoid overestimating the overall accuracy [31].

**Figure 1:**
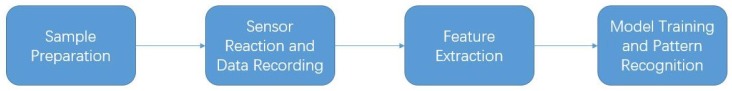
Research steps.

Based on previous research and analysis, our group selected 12 different categories of typical herbal medicines used in China, including Astragalus, Liquorice, and Chinese Angelica, as the medicines for the experiments. Using a self-assemblied electronic nose system, our group used support vector machine (SVM) [32,33], decision tree (DT) [34], linear discriminant analysis (LDA) [35], K-nearest neighbors (KNN) [36], artificial neural network (ANN), Naive Bayes (NB), principal component analysis (PCA) and conformal prediction based on K-nearest neighbors (CP-KNN) to distinguish between and analyze diverse herbal medicines with leave-one-out cross validation. The whole process can be interpreted from Figure 1.

## 2. Conformal Prediction

### 2.1. Definition

Classification and regression are the usual issues in machine learning. Herbal medicine discrimination is one of the classification problems. Generally, in classification problems, there is usually a training set with many observations containing features and labels: $\left(\left(x_{1},y_{1}\right),...,\left(x_{n},y_{n}\right)\right)$.

Each observation consists of one feature vector ($x_{i}\in X$) and one label ($y_{i}\in Y$) in which *X* is the object space and *Y* is the label space. As long as the objects and labels are established, example space *Z* can be established:

(1) $$z_{i}=\left(x_{i},y_{i}\right),i=1,2,...,\left(n-1\right).$$

When a new sample ($x_{n}$) appears, the task is to predict the label ($y_{n}$) associated with it.

A simple predictor finds one function (F) which reflects the new feature vector ($x_{n}$) in object space to one label in the label space:

(2) $$F:Z^{*}\times X⟶Y.$$

A conformal predictor has another parameter, the significance level (ϵ∈(0,1)). Additionally, the confidence level, representing the confidence underlying each prediction, is represented by 1−ϵ. Given a specific significance level, a conformal predictor outputs a set of predicted labels in the label space based on the conformity of each label predicted:

(3) $$\Gamma^{\epsilon }\left(z_{1},...,z_{n-1},x_{n}\right).$$

Meanwhile, the output sets from conformal predictors are nested as shown below:

(4) $$\Gamma_{1}^{\epsilon_{1}}\left(z_{1},...,z_{n-1},x_{n}\right)\subset \Gamma_{2}^{\epsilon_{2}}\left(z_{1},...,z_{n-1},x_{n}\right)\left(\forall \epsilon_{1}\geq \epsilon_{2}\right).$$

Conformal predictors output prediction sets according to the nonconformity measure, which can be represented by matrix *A* that associates each observation ($z_{i}\in Z\left(i=1,2,\ldots ,n\right)$) with a real number ($\alpha_{i}\in R\left(i=1,2,\ldots ,n\right)$). This nonconformity measure, based on a specific algorithm such as KNN, indicates how well conformed each combination of feature and sample is when placed in the observations excluding the observation being examined:

(5) $$\alpha_{i}=A_{n}\left(z_{1},\ldots z_{i-1},z_{i+1},\ldots ,z_{n}\right),i=1,\ldots ,2.$$

Additionally, *A* has exchangeability; for any n and any permutation (π),

(6) $$\left(\alpha_{1},\ldots ,\alpha_{n}\right)=A_{n}\left(z_{1},\ldots ,z_{n}\right)⟶\left(\alpha_{\pi (1)},\ldots ,\alpha_{\pi \left(n\right)}\right)=A\left(z_{\pi (1)},\ldots ,z_{\pi (n)}\right).$$

After giving the definition of nonconformity measure, the conformal predictor dependent on *A* can be represented by:

(7) $$\Gamma^{\epsilon }\left(z_{1},z_{2},\ldots z_{n-2},z_{n-1},z_{n}\right)=\left\{y|p^{y}>\epsilon \right\}.$$

For a newly emerged feature vector ($x_{n}$), a conformal predictor evaluates the conformity level of each combination of the vector and every possible label in the label space, which is represented by the *p*-value given below. For each possible label (y∈Y), the *p*-value associated with it is defined as:

(8) $$p^{y}=\frac{\left|\{i=1,\ldots ,n|\alpha_{i}^{y}>\alpha_{n}^{y}\}\right|}{n},$$

where $p^{y}$ represents how well the newly added observation conforms to the existing observations when the label of $x_{n}$ is *y*.

Then, it outputs all the possible labels in the set $\Gamma^{\epsilon }$ based on a given significance level (ϵ∈(0,1)).

For conformal prediction, the validity of the prediction means:

(9) $$P(y_{n}\in \Gamma^{\epsilon }\left(x_{1},y_{1},\ldots ,x_{n-1},y_{n-1},x_{n}\right))>1-\epsilon ,$$

(10) $$P(y_{n}\notin \Gamma^{\epsilon }\left(x_{1},y_{1},\ldots ,x_{n-1},y_{n-1},x_{n}\right))<\epsilon .$$

### 2.2. Nonconformity Measure

In theory, any algorithm can be modified to measure nonconformity. Vladimir Vovk initially used KNN as the fundamental algorithm. Therefore, this typical method was also implemented in our work. For sake of simplicity and conciseness, we used CP-1NN and CP-3NN to represent conformal prediction based on 1NN and 3NN respectively. To use KNN to calculate nonconformity measure for a feature-label combination $\left(x_{i},y_{i}\right)$, to begin with, the distances between this combination and any other observation already in training set were calculated and denoted as:

(11) $$d(x_{i},x_{j}),j=1,2,\ldots ,i-1,i+1,\ldots ,n.$$

Then, *k* nearest observations sharing the same label were found: $\left(x_{is},y_{is}\right)$, s=1,…,k. Meanwhile, *k* nearest observations with different labels were found: $\left(x_{js},y_{js}\right)$, s=1,…,k. Afterwards, the nonconformity measure was calculated with the following formula:

(12) $$\alpha_{i}=\frac{\sum_{s=1}^{k}d\left(x_{is},y_{is}\right)}{\sum_{s=1}^{k}d\left(x_{js},y_{js}\right)}.$$

From what has been shown, a corollary can be made that the closer to observations sharing the same label the new combination is, the better conformed the combination is, which indicates a higher confidence for the feature–label combination.

### 2.3. Offline Conformal Prediction

With the nonconformity measurement mentioned above, offline conformal predictors, which are based on fixed training set and test sets, can inform users of the confidence and credibility of each prediction which enables users to make decisions more wisely based on the reliability of each prediction. Conformal predictors can be required to output the label with the highest *p*-value, which is referred to as forced prediction [31]. Along with the predicted results, conformal predictor shows confidence and credibility:

(13) $$confidence=sup\{1-\epsilon :\;|\Gamma^{\epsilon }|\leq 1\},$$

(14) $$credibility=inf\{\epsilon :\;|\Gamma^{\epsilon }|=0\}.$$

In the classification, the confidence score is equal to 1 minus the second largest *p*-value which indicates the confidence of rejecting other possible labels. Credibility is equal to the largest *p*-value which shows how good the output label is in terms of conformity. Based on this measure, a reliable prediction has a confidence of approximately 1 and a credibility of not too close to 0.

## 3. Experiments and Data Processing

### 3.1. Medicine Selection and Preprocessing

**Figure 2:**
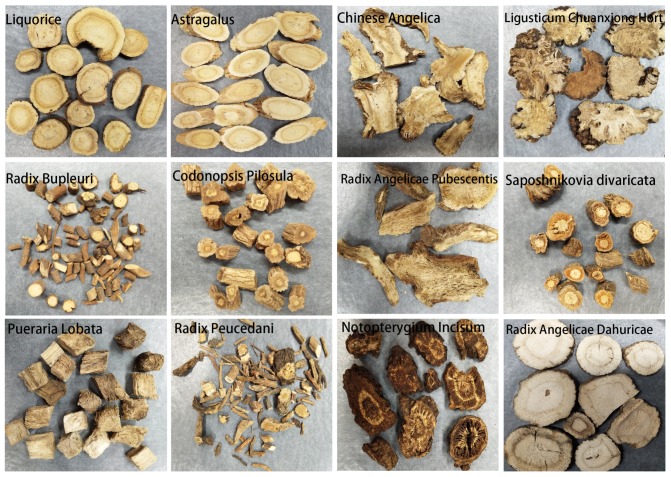
Physical appearances of the 12 categories of herbal medicine.

Based on research, our group selected 12 different categories of traditional Chinese herbal medicines for experiments (including Astragalus, Liquorice, Chinese Angelica, Saposhnikovia Divaricata, Radix Angelicae Pubescentis, Radix Angelicae Dahuricae, Notopterygium Incisum, Codonopsis Pilosula, Radix Bupleuri, Ligusticum Chuanxiong Hort, Radix Peucedani, and Pueraria Lobata). These herbal medicines mainly come from the Umbelliferae Apiaceae family. In addition, all the medicines are derived from the root parts of each category. The criteria of our selection were the similarities between their appearances and biological classifications, and the misleading and substituting phenomena that appear in the market. The physical appearances of the medicines are shown in Figure 2.

We firstly used an electric pulverizer to grind the 12 kinds of medicines listed above into powders. Then, we prepared 50 samples for each kind of medicine (a total of 12 × 50 = 600 samples). We gathered 8 g of powder into 125 mL glass containers, sealed the containers with para-films, heated the samples for 10 h in an incubator whose temperature was set to be 50 ${}^{\circ }$C, and waited while the volatile gases above the powder were saturated for 10 h.

### 3.2. Self-Assembled Electronic Nose System and Experiment

**Figure 3:**
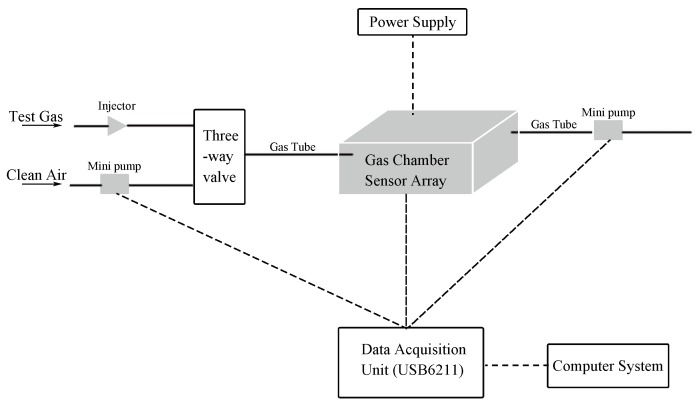
Layout of self-assembled E-nose system.

The equipment used was an electronic nose system from the State Key Laboratory of Industrial Control Technology in Zhejiang University [31,37,38], which contains 16 TGS (Taguchi Gas Sensors) type metal oxide semi-conductive (MOS) sensors bought from Figaro Engineering Inc. (Osaka, Japan). TGS sensors have already shown robust performances in food classification [39,40]. Each sensor in this sensor panel fixed on a circuit in a 200 mL chamber has respective affinity while reacting with certain gases and the features are listed in Table 1. The sensors selected can respond to the volatile gas compounds emanated by different types of herbal medicines which includes many different types of phenols and aldehydes. Meanwhile, these sensors do not have excessive specificity towards one type of gas which is appropriate for herbal medicine odors which have complicated compositions. A heater voltage of 5 V was supplied to each sensor to ensure better performance in accordance with the recommendations from Figaro Engineering Inc. A brief layout of the system is shown in Figure 3.

The gas transporting system consisted of a three-way valve and two gas pumps to alter between the flow of target gas and the flow of standard gas (clean and dry air). Meanwhile, the pump system ensured that the flow of gas was 1 L per minute. With regard to the signal recording system, a data acquisition (DAQ) unit USB 6211 manufactured by National Instrument (Austin, TX, USA) was bought to record the responses of sensors and the control signals of valves and pumps using a software based on Labview 2014.

**Figure 4:**
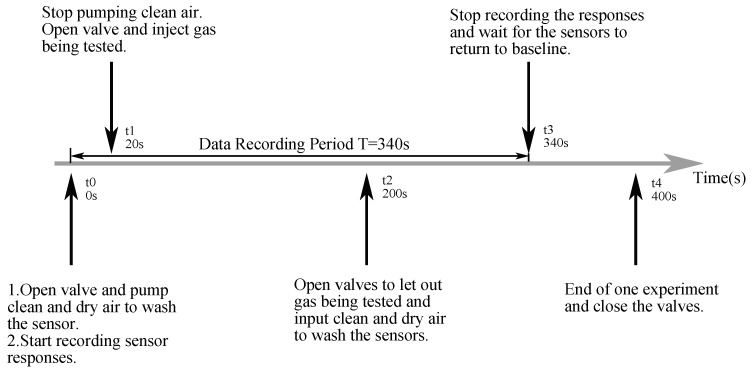
Experiment process for one single sample.

We used the electronic nose system to analyze all 600 samples, one by one. The environment temperature was 22–27 ${}^{\circ }$C and the relative humidity in the laboratory during experiments was 50–70% and the environment conditions were relatively stable during experiments. The overall process of the experiment for one single sample is shown in Figure 4. Each individual test lasted for 400 s in total and the sampling frequency was set to be 100 Hz. Firstly, sensor panels were cleaned with 1 L/min standard gas to return to the baselines for 20 s. Secondly, target gas was filled into the chamber and the flow of standard gas was stopped. We used medical injectors to extract 10 mL gas mixtures in the head-space of each sample and injected the gas into the chamber where the sensor panel was located. The injection of target gas was less than 1 s and the gas was injected as soon as possible. Afterwards, a period of 180 s was set for the reaction which was regarded as the “rising and stabilizing” period, after which the standard gas was pumped in again and the pump letting gas out was also opened. The recording process lasted for another 140 s after the system entered the “declining” period. Finally, the sensors were stabilized for another 60 s.

### 3.3. Data Processing and Feature Extraction

**Figure 5:**
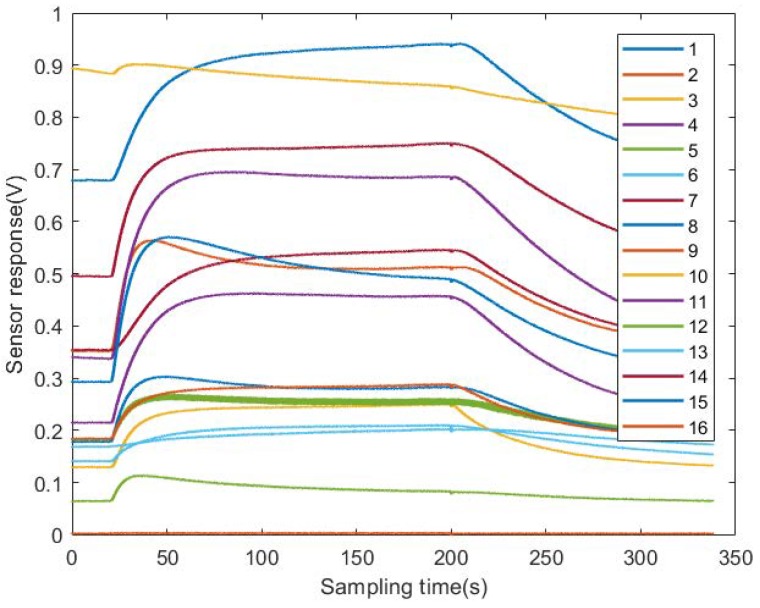
Sensor responses to Astragalus given by the E-nose system (voltage (v) versus time (0.01 s)).

Typical sensor response curves are shown in Figure 5; these indicate the responses to the sixth Astragalus sample. Firstly, all the data from the sensor signals were calibrated by subtracting the baselines to eliminate sensor drifts:

(15) $$V=V_{S}-V_{0},$$

where $V_{s}$ is the actual response of sensors, and $V_{0}$ represents the baseline value.

Afterwards, 8 commonly used features for each sensor (a total of 8 × 16 = 128 features) were extracted:

1. Maximum Value
  (16) $$V_{max}=max\left(|V|\right).$$
2. Integral Value
  (17) $$V_{int}=\int_{0}^{T}V\left(t\right)dt,$$
  where T represents the total time for one record (T = 340 s).
3–8. Exponential moving average of the derivative of V [41]
  (18) $$E_{a}\left(V\right)=\left[min\left(y\left(k\right)\right),max\left(y\left(k\right)\right)\right],\;2000<k<34,000.$$

The discrete sampling exponential moving average and smoothing factor *a* were defined as

(19) y(k)=(1−a)y(k−1)+a(V(k)−V(k−1)),

(20) $$a=\frac{1}{100*SR},\frac{1}{10*SR},\frac{1}{SR},$$

(21) y(1)=aV(1),

where SR=100 represents the sampling frequency. $E_{a}\left(V\right)$ is the vector containing the smallest and largest values in the period of time after the injection of the gas being tested. After feature extraction, feature matrices (600 × 128) were made for the 12 herbal medicines. Data processing was conducted by MATLAB R2016a and R2017b.

## 4. Results and Discussion

### 4.1. Performances of Simple Predictors

Using the leave-one-out cross validation method, we used seven different kinds of classification algorithms as our simple predictors in the classification task. The results are listed in Table 2 with parameters tuned for each algorithms. For instance, performances of SVM with different kernels and KNN with different k parameters are shown in Table 3 and Table 4. As is indicated in this table, in this classification problem, SVM performed the best with a classification accuracy of 98.94%, and LDA was second with an accuracy of 98.33%. Meanwhile, despite the differences in their respective classification accuracies, all 7 algorithms classified these 12 categories of herb medicines with accuracies above 90% which justifies the use of the electronic nose and machine learning algorithms in herbal medicine classification.

### 4.2. PCA Analysis

For classification algorithms, accuracy is not the solitary factor that should be taken into consideration. Additionally, the time cost of classification should be taken into consideration. In order to show the time cost and to lower time cost, we introduced the principal component analysis (PCA) which is a frequently used algorithm that is used to reduce the feature dimensionality with the least amount of sacrifice. In this section, the classification accuracy and time cost measured by PCA for the six specific algorithms used in the classification are mentioned. The results are illustrated in Table 5, and the computer we have used was a normal personal computer (CPU: Intel Core i7-7700HQ @ 2.80 GHZ, 4 Cores; RAM: DDR4 2400 MHz 8 GB, MSI, New Taipei City, Taiwan). For this analysis, we calculated the mean accuracy and mean time spent on five runs of each algorithm.

Based on the results shown in the table, it is clear that, generally, with lower dimensions, the classification accuracy shows a declining tendency. What is more, with reduced dimensionality, it takes far less time for each algorithm to model and classify all 600 samples which justifies the meaning of PCA. When comparisons are made among different algorithms, SVM and LDA tend to outperform other algorithms in terms of accuracy when the dimensions are 128 or 30, but a greater amount time must be sacrificed. However, with the reduction of dimensions, this accuracy advantage tends to be less evident. Therefore, according to this analysis, it is advisable for users to choose classification algorithms wisely and strike a balance between accuracy and time cost by using PCA, a great tool for reducing feature dimension.

**Figure 6:**
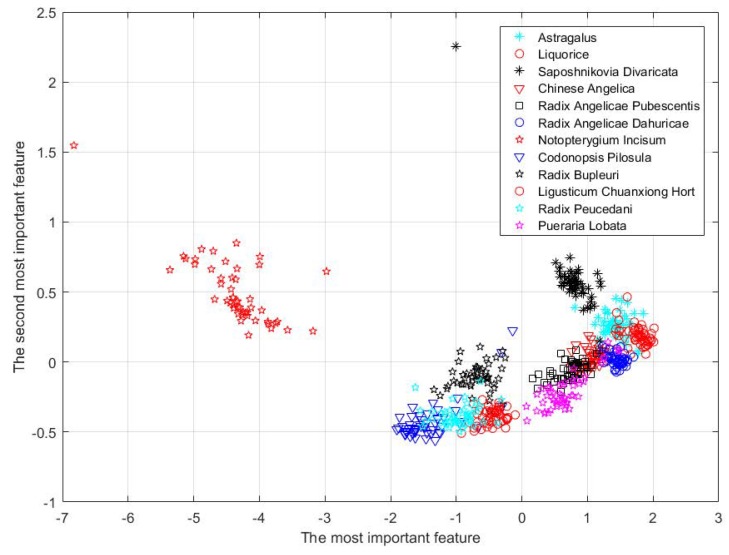
Distribution of samples after PCA.

Meanwhile, with PCA, we can depict the distribution of samples by lowering the number of dimensions to 2D, as is shown in Figure 6. From this figure, we can see that, visually, it is possible for different kinds of herbal medicines to be classified even if we condense the features into a 2D space.

### 4.3. Performance of Conformal Prediction

In addition to the machine learning algorithms mentioned above, to gain more information about the prediction reliability, we applied conformal predictions based on 1NN (CP-1NN) and 3NN (CP-3NN) in our work. The prediction accuracies in offline mode are listed in Table 6. According to the accuracy results, it is clear that CP-1NN performed worse when compared with the simple predictor 1NN, while CP-3NN performed better when compared with the simple predictor 3NN. Meanwhile, CP-3NN performed better than CP-1NN in terms of accuracy in our experiments.

**Figure 7:**
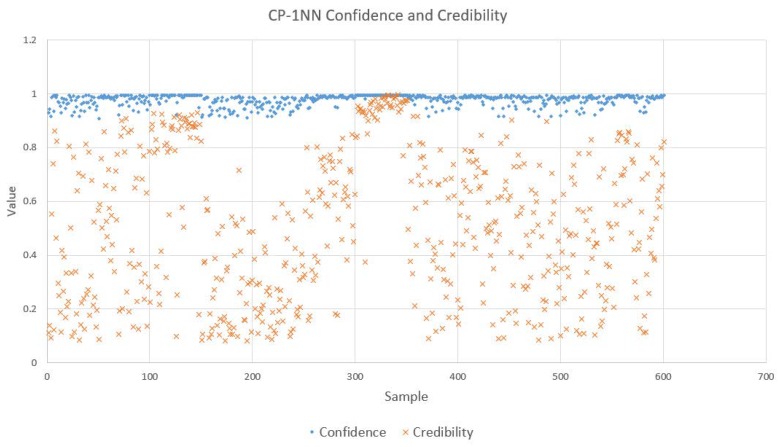
Confidence values and credibility levels for 12 herbal medicine classifications with CP-1NN.

**Figure 8:**
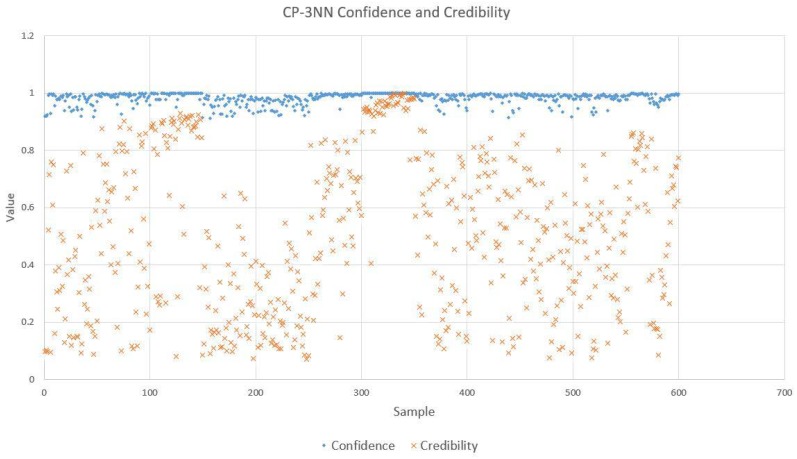
Confidence and credibility for 12 herbal medicine classifications with CP-3NN.

For conformal predictors, predictions are not the only products. What is also significant is the reliability information, which is given as confidence and credibility. We randomly selected 5 samples from a total of 600 samples which used CP-1NN for analysis to serve as examples, as is shown in Table 7. According to this table, CP-1NN gave correct labels for the samples with indices of 5, 233, and 512, and the confidence values associated with them were approximately 1, while their respective credibility levels did not approximate 0, which manifests the high prediction reliability. On the contrary, CP-1NN erroneously predicted the labels for sample 384 and sample 478. These two predictions have high confidence but low credibility, which justifies the wrong outputs of CP-1NN. After analyzing these five examples, we depicted the confidence and credibility levels of all 600 predictions with CP-1NN and CP-3NN, as shown in Figure 7 (CP-1NN) and Figure 8 (CP-3NN). We can see that nearly all predictions tend to feature high confidence but credibility values vary with diverse samples. Through comparing CP-1NN and CP-3NN, we found that predictions given by CP-3NN showed higher confidence values but lower credibility levels. A possible reason for this may be that with the addition of new neighbors for nonconformity measurement, confidence can be boosted with more information about the sample space. Meanwhile, when a larger number of neighbors is taken into consideration, the model can be more complicated and the requirement for conformity of each single feature–label combination depends not on one sample but three samples which may cause the credibility to suffer.

With the additional reliability information given by conformal predictors, users can gain a better understanding of each prediction and make better decisions based on the classification results.

### 4.4. Implications and Discussions

As shown in the results above, by using electronic nose, a convenient and non-instrumental analyzing approach, we managed to effectively discriminate between 12 different categories of herbal medicine. Additionally, accuracy varied with different algorithms, and it was shown that PCA can be used to lower the time expenditure in discrimination and to visualize the sample distribution on a 2D plane. This work provides great insights into the construction of an alternative herbal medicine flavor fingerprint library which would aid in the identification of inferior substitutes that have appeared in herb medicine markets, leading to better treatment for patients. Meanwhile, since, in addition to prediction accuracy, reliability also plays an important role in medicine evaluation tasks, as has been shown in previous research [31], we used conformal prediction and provided a method of revealing the prediction reliability. This enables users to understand the potential risks. When conformal predictors indicate low reliability, users can be advised to further analyze the medicines with other methods, which lowers the possibility of misjudgement solely based on electronic nose when the results are not reliable. This additional reliability information given by conformal prediction enables practitioners to evaluate herbal medicine qualities more effectively. If the machine gives out an unreliable prediction result, it is advisable for users to re-examine the medicine with other analyzing measures, contributing to more faith and credibility in the market.

Admittedly, analysis with the electronic nose only still has disadvantages. There are several things that should be taken into consideration while constructing flavor fingerprint library.

Firstly, sensor driftt, sensor poisoning, and sensitivity variation are common problems associated with the E-nose, as mentioned in previous publications [31,42]. Therefore, data fusion with data gathered from other analytical means, such as electronic tongue and spectroscopy, could be a promising solution to allow better accuracy and credibility, as shown in previous studies [25,31].

Secondly, the detailed and concrete relationships between volatile compound gases from herbal medicines and sensor responses have not been fathomed by researchers. So far, there is no universal E-nose that is suitable for all applications, as mentioned by Ref. [6]. Therefore, if the physical basis for analysis with electronic nose is determined by more scholars, a specifically-made electronic noses targeted at alternative herbal medicine discrimination could be invented, significantly reducing the cost of classification.

What is more, in this work, we selected eight features for each sensor. More studies on areas such as sensor selection and optimization [37] should be done in the future to improve the feature extraction process to solve problems such as superfluous information and sensitivity declines.

Finally, as shown in this work, different algorithms tend to have different levels of performance. So far, scholars have used different algorithms when applying the electronic nose in their respective domain, s and there has been no standard algorithm that has been vastly agreed on for discriminating between herbal medicines with the electronic nose [5,9,23,25,31]. Therefore, it is necessary that more research on the choice of pattern recognition algorithms that are most suitable for electronic nose application are done to find out the most appropriate algorithms based on real-world data.

## 5. Conclusions

In this work, after careful selection and experiments, discrimination analysis of 12 categories of herbal medicines were done with a self-assemblied electronic nose system. Though different in their performances, seven algorithms managed to classify the 12 categories of heral medicines, with SVM and LDA outperforming other algorithms with the highest accuracies of 98.94% and 98.33% respectively. After using PCA, the time expenditure required for classification can be reduced; however, it is also possible that the accuracy may decrease, and it is inevitable for users to balance between time cost and classification accuracy. Among all seven algorithms, the conformal predictions based on KNN (CP-1NN and CP-3NN) can give additional and significant information about the prediction reliability without sacrificing accuracy, which is of key importance, since medicine quality is closely associated with patients’ health and treatment. This essay provides an inspirational approach for the establishment of a herbal medicine flavor fingerprint library which could facilitate the quick and accurate recognition and evaluation of herbal medicines.

## Figures and Tables

**Table 1:** The response characteristics of sensors.

No.	Sensor Type	Specific Response Sensitivity
1	TGS800	Carbon monoxide, ethanol, methane, hydrogen, ammonia
2	TGS813	Carbon monoxide, ethanol, methane, hydrogen, isobutane
3	TGS813	Carbon monoxide, ethanol, methane, hydrogen, isobutane
4	TGS816	Carbon monoxide, ethanol, methane, hydrogen, isobutane
5	TGS821	Carbon monoxide, ethanol, methane, hydrogen
6	TGS822	Carbon monoxide, ethanol, methane, acetone, n-hexane,
		benzene, isobutane
7	TGS822	Carbon monoxide, ethanol, methane, acetone,
		n-Hexane, benzene, isobutane
8	TGS826	Ammonia, trimethyl amine
9	TGS830	Ethanol, R-12, R-11, R-22, R-113
10	TGS832	R-134a, R-12 and R-22, ethanol
11	TGS880	Carbon monoxide, ethanol, methane, hydrogen, isobutane
12	TGS2620	Methane, Carbon monoxide, isobutane, hydrogen
13	TGS2600	Carbon monoxide, hydrogen
14	TGS2602	Hydrogen, ammonia ethanol, hydrogen sulfide, toluene
15	TGS2610	Ethanol, hydrogen, methane, isobutane/propane
16	TGS2611	Ethanol, hydrogen, isobutane, methane

**Table 2:** Classification performances of different algorithms.

Prediction Tasks and Algorithms	DT	KNN	LDA	SVM	NB	BP (Back Propagation)
12 Categories of herbal medicine	92.17%	91.67%	98.33%	98.94%	91.33%	90.83%

**Table 3:** Prediction accuracy of SVM with different kernels in offline mode.

Task and SVM Kernel	Linear	Quadratic	MLP (Multilayer Perceptron Kernel)	RBF (Radial Basis Function)
12 TCM discrimination	98.94%	98.92%	82.51%	93.69%

**Table 4:** Prediction accuracy of KNN with parameter k in offline mode.

The K of KNN	1	3	5	7	9
12 TCM discrimination	91.67%	91.50%	90.17%	90.00%	88.50%

**Table 5:** PCA analysis in terms of accuracy and time cost.

**Test Item**	**DT**	**1NN**	**3NN**	**LDA**	**SVM**	**NB**
Accuracy	92.17%	91.67%	91.50%	98.33%	98.94%	91.33%
Time(s)	36.605	0.277	0.293	37.987	967.555	166.992
**PCA:30-D (99.74% Information)**	**DT**	**1NN**	**3NN**	**LDA**	**SVM**	**NB**
Accuracy	81.83%	91.17%	90.67%	95.50%	97.64%	87.50%
Time(s)	15.208	0.122	0.152	31.759	695.299	48.531
**PCA:5-D (95.44% Information)**	**DT**	**1NN**	**3NN**	**LDA**	**SVM**	**NB**
Accuracy	82.33%	87.67%	87.67%	85.00%	87.32%	84.50%
Time(s)	6.984	0.081	0.084	29.778	252.202	17.679

**Table 6:** Conformal prediction accuracy in offline mode.

Prediction Tasks	CP-1NN	CP-3NN	1NN	3NN
12 categories of herbal medicines	91.50%	92.17%	91.67%	91.50%

**Table 7:** Five typical individual predictions for 12 herbal medicine classifications with CP-1NN.

Sample Index	True Label	Forced Prediction	Confidence	Credibility
5	1 (Astragalus)	1 (Astragalus)	0.9950	0.7433
233	5 (Radix Angelicae Pubescentis)	5 (Radix Angelicae Pubescentis)	0.9883	0.4650
384	8 (Codonopsis Pilosula)	10 (Ligusticum Chuanxiong Hort)	0.9400	0.1317
478	10 (Ligusticum Chuanxiong Hort)	8 (Codonopsis Pilosula)	0.9183	0.0867
512	11 (Radix Peucedani)	11 (Radix Peucedani)	0.9950	0.7383

## Abbreviations

The following abbreviations are used in this manuscript:

DT	Decision Tree
NB	Naive Bayes
SVM	Support Vector Machine
LDA	Linear Discriminant Analysis
PCA	Principal Component Analysis
KNN	K-Nearest Neighbors
CP	Conformal Prediction
TCM	Traditional Chinese Medicine
